# A novel scale based on biomarkers associated with COVID-19 severity can predict the need for hospitalization and intensive care, as well as enhanced probabilities for mortality

**DOI:** 10.1038/s41598-023-30913-4

**Published:** 2023-06-04

**Authors:** Eduardo Nieto-Ortega, Alejandro Maldonado-del-Arenal, Lupita Escudero-Roque, Diana Ali Macedo-Falcon, Ana Elena Escorcia-Saucedo, Adalberto León-del-Ángel, Alejandro Durán-Méndez, María José Rueda-Medécigo, Karla García-Callejas, Sergio Hernández-Islas, Gabriel Romero-López, Ángel Raúl Hernández-Romero, Daniela Pérez-Ortega, Estephany Rodríguez-Segura, Daniela Montaño‑Olmos, Jeffrey Hernández-Muñoz, Samuel Rodríguez-Peña, Montserrat Magos, Yanira Lizeth Aco-Cuamani, Nazareth García-Chávez, Ana Lizeth García-Otero, Analiz Mejía-Rangel, Valeria Gutiérrez-Losada, Miguel Cova-Bonilla, Alma Delia Aguilar-Arroyo, Araceli Sandoval-García, Eneyda Martínez-Francisco, Blanca Azucena Vázquez-García, Aldo Christiaan Jardínez-Vera, Alejandro Lechuga-Martín del Campo, Alberto N. Peón

**Affiliations:** 1 Sociedad Española de Beneficencia, Av. Juárez #908, Col. La Villita, CP42060, Pachuca, Hidalgo Mexico; 2 Área Académica de Medicina, Universidad Autónoma del Estado de Hidalgo, Pachuca, Mexico; 3 Escuela Superior de Apan, Universidad Autónoma del Estado de Hidalgo, Carretera Apan‑Calpulalpan s/n, Colonia, 43920 Chimalpa Tlalayote, Hgo Mexico; 4 Universidad Anáhuac, Puebla, Mexico; 5 Hospital Español, Blvd. Luis Donaldo Colosio 802, El Palmar, Pachuca, Mexico; 6 Facultad de Medicina, Universidad Nacional Autónoma de México, Av. Universidad 3004, Copilco Universidad, Coyoacán, 04510 Mexico City, Mexico; 7 Servicio de Imagenología, Hospital Regional de Alta Especialidad de Zumpango, Carretera Zumpango-Jilotzingo 400 Barrio de Santiago 2a Seccion, 55600 Zumpango de Ocampo, Estado de México Mexico

**Keywords:** Health care, Medical research, Risk factors

## Abstract

Prognostic scales may help to optimize the use of hospital resources, which may be of prime interest in the context of a fast spreading pandemics. Nonetheless, such tools are underdeveloped in the context of COVID-19. In the present article we asked whether accurate prognostic scales could be developed to optimize the use of hospital resources. We retrospectively studied 467 files of hospitalized patients after COVID-19. The odds ratios for 16 different biomarkers were calculated, those that were significantly associated were screened by a Pearson’s correlation, and such index was used to establish the mathematical function for each marker. The scales to predict the need for hospitalization, intensive-care requirement and mortality had enhanced sensitivities (0.91 CI 0.87–0.94; 0.96 CI 0.94–0.98; 0.96 CI 0.94–0.98; all with *p* < 0.0001) and specificities (0.74 CI 0.62–0.83; 0.92 CI 0.87–0.96 and 0.91 CI 0.86–0.94; all with *p* < 0.0001). Interestingly, when a different population was assayed, these parameters did not change considerably. These results show a novel approach to establish the mathematical function of a marker in the development of highly sensitive prognostic tools, which in this case, may aid in the optimization of hospital resources. An online version of the three algorithms can be found at: http://benepachuca.no-ip.org/covid/index.php

## Introduction

Two years and ten months after the SARS-CoV-2 pandemics started, more than 621 million cases and more than 6.54 million COVID-19-related deaths have been documented by the World Health Organization (WHO)^1^. In such a rapid growing outbreak hospital resiliency has been repeatedly challenged worldwide^2^, imposing an important toll on the physical and mental health of healthcare workers^3,4^, and leading to the saturation of regular hospital beds as well as intensive care unit (ICU) beds, thus producing a reduction on the quality of medical attention^5^, which may have impacted in an enhanced mortality, not only in SARS-CoV-2 patients, but on the totality of cases.

Moreover, in some developing countries healthcare centers reached a complete saturation by COVID-19 patients, leaving many SARS-CoV-2 positive and negative patients without a hospital bed^6,7^. Arguably, many of the coronavirus-related hospitalizations were unnecessary, and those patients could have been treated at home, freeing hospital resources for patients with enhanced needs. Nonetheless, a precise system to discriminate such cases in order to optimize hospital resource use is lacking.

On the other hand, while more than 80% of COVID-19 patients have a mild to asymptomatic disease, 20% of the patients present moderate to critical forms of the illness^8^, and mortality ranges from less than 1–5.4%. While some drugs may prevent the progression to critical illness and mortality, their use should be timely^9,10^, therefore the development of a prognostic tool with enhanced ease of use, speed and accuracy may be of paramount importance to prevent such outcomes.

In this scenario, a series of prognostic scales have been developed to predict either the potential for mortality or for aggravating disease, but they are not accurate^11,12^, are published in non-peer-reviewed journals^13^, are developed after small samples^14^, are not COVID-19-specific^15^ or only predict mortality^16^; therefore many clinical practitioners may had the impression that these prognostics could be done easily and accurately, perhaps leading to the aggravation of the problem. We think that in order to enhance the capacity of healthcare workers to optimize the use of hospital resources, including regular and ICU beds, or the administration of life-saving drugs that are either expensive and/or scarce, three prognostic tools should be developed: one to predict the need for hospitalization, another to predict the need for intensive care (IC), and lastly one to predict the potential for mortality.

In the present article we show the development of a prognostic algorithm that is able to predict these outcomes specifically in COVID-19 patients. The tool was developed after the study of 422 patients and shows an enhanced sensitivity (over 90%). Moreover, we made a confirmatory study with the patients of a different hospital, finding that the sensitivity did not change considerably. An online, easy to use, version of this tool can be found on: http://benepachuca.no-ip.org/covid/index.php

Finally, the methodological approach to establish the mathematical functions of the different biomarkers by the means of the Pearson’s correlation index is, to our knowledge, novel. And we think that may aid in the development of similar prognostic tools.

## Materials and methods

We gathered a total of 467 clinical files belonging to patients that were hospitalized at Sociedad Española de Beneficencia and Hospital Español, from March 12th 2020 to August 1st 2022 with the diagnosis of COVID-19. The records were screened for the following inclusion criteria: (i) patients with confirmed SARS-CoV-2-derived infection (positive PCR, rapid antigen, IgM and/or CORADS 4 or higher), (ii) files that showed laboratory evaluations in the first 24 h after hospital admission, (iii) patients with signed informed consent for the study. Then the records that complied with one or more of the following exclusion criteria were eliminated: (i) voluntarily-discharged patients, (ii) patients with clinical files lacking crucial information, (iii) post-COVID-19 care, and (iv) patients that were referred from other institutions and therefore had incomplete records.

Relevant data about markers that were previously associated with severe COVID-19 were collected on a Google Docs file, sorting the patients according to three different outcomes: mortality, intensive care requirement (ICR) and hospitalization requirement (HR). Mortality was defined as death occurring within hospitalization; ICR was defined as the use of the ICU staff and facilities for at least one day; and HR was defined as patients requiring a respiratory support superior to 5 L/min for at least one day (because this requirement is most likely unsustainable in an *at home* treatment setting).

The control patients for HR, ICR or mortality were those that did not required more than 10L/min of oxygen supplementation, intensive care or did not died within the hospital, respectively. The percentage of lung infiltration was evaluated as described elsewhere^17–19^ using the Chest CT Score. Briefly, chest computed tomography studies were evaluated by two independent researchers whom divided the lungs into five anatomical regions (one for each lobule), and assigned each one up to five points depending on the percentage of the parenchyma that was infiltrated, adding the points at the end to a maximum of 25 points.

The level of association of each marker with the three different outcomes was initially assessed by calculating the odds ratio (OR), and the markers were considered to have a significant association with a particular outcome when *p* ≤ 0.05. For this test the reference values were those published elsewhere^20^. We then performed a Kolmogorov–Smirnov test in order to determine the type of distribution of the data (data not shown). Then, the patients’ values were transformed in a binary manner, considering their value as “0” when they did not surpassed their reference values, and as “1” when they did. The binary data for significant associated markers was used to perform a Pearson’s correlation to estimate their weight or mathematical function, but only those that had a Pearson’s correlation index ≥ 0.20 were integrated into the pertinent algorithm. These consisted on the addition of the function of each marker (Pearson’s index) when the patient exceeded the marker’s reference values. Importantly, being that our data followed a non-Gaussian distribution, we opted for the Sperman’s correction for the Pearson’s test.

The individual patient’s outcome predictor (OP) values were calculated and plotted into a Receiver Operating Characteristic (ROC) curve to estimate the sensitivity of each algorithm in the prediction of the aforementioned outcomes. Furthermore, the mean and standard deviation of the control group (negative for each outcome) were added, while the standard deviation was deducted from the mean of the outcome-positive group, and the middle point between each operation’s results was found to calculate the cutoff value. Sensitivity (SE), specificity (SP), positive (PPV) and negative predictive (NPV) values, as well as the OR and Chi^2^ values were then calculated to investigate each algorithm’s characteristics. All the statistic tests were performed and graphed using GraphPad Prism X9, and significant differences were considered when *p* ≤ 0.05.

A protocol for this study was evaluated by the Institutional Committee of Research Ethics of the Sociedad Española de Beneficencia (Pachuca, Hidalgo) and the study was approved on February 24th of 2020. Our sponsor had no role in study design. All methods were performed in accordance with the relevant guidelines and regulations, including the Declaration of Helsinki, and written informed consent was obtained from all the patients studied.

## Results

We assessed a total of 467 clinical files belonging to patients that were hospitalized at Sociedad Española de Beneficencia and Hospital Español, from March 12th 2020 to August 1st 2022. All files were analyzed, and 422 were found to be suitable for analysis. 255 files were allocated to algorithm design and 167 were used to validate the algorithms (Supp. Fig. S1). The only criteria for such allocation was to use the files belonging to Hospital Español in the design of the algorithms, while the files from Sociedad Española de Beneficencia were used to validate the tools with a different population. The patients whom contributed with the data for algorithm design were unvaccinated against SARS-CoV-2, while only 26% of the patients that provided data for the algorithms’ validation had already received such treatment.

Of the 255 clinical files that we used to calculate the algorithm 175 (74.5%) belonged to patients that were retrospectively found to have a justifiable hospitalization, while 59 (25.5%) did not develop characteristics that made hospitalization mandatory over their whole hospital stay. Moreover, 125 (49.6%) patients required IC and 79 (31.1%) died at the hospital.

On the other hand, given that severely affected chest tomography findings (% inf)^21^, C-reactive protein (CRP), d-dimer, neutrophils, lymphocytes, lactate dehydrogenase (LDH)^22^, procalcitonin, medium arterial pressure (MAP), creatinine, leukocytes, aspartate aminotransferase (AST)^23,24^, ferritin, oxygen saturation (sO_2_)^25^, and advanced age and comorbidities^26^ have been associated with COVID-19 progression, the exact values of these markers were extracted from the complete clinical files of the participants. Upon gathering the laboratory data for the first 24 h of hospitalization, the OR for each of these markers was calculated in relation to HR (Supp. Table S1), ICR (Supp. Table S2) or enhanced probability for mortality (Supp. Table S3).

We found six significant associated markers with HR, which were a Kirby index < 300 (OR 5.58, CI 1.95–15.97, *p* = 0.0010) and Kirby index < 200 (OR 34.16, CI 15.10–70.99, *p* = 0.0001), as well as sO_2_ < 90% (OR 2.15, CI 1.138–3.30, *p* = 0.0133), sO_2_ < 80% (OR 3.04, CI 1.415–6.833, *p* = 0.0043), CRP > 120 mg/dL (OR 2.95, CI 1.439–5.838, *p* = 0.0021) and LDH > 400 U/L (OR 2.6, CI 1.393–4.982, *p* = 0.0034).

Moreover, we found 13 significant association with ICR, which were d-dimer > 500 ng/mL (OR 2.411, CI 1.160–5.017, *p* = 0.0179), neutrophils > 7700 cells/µL (OR 1.807, CI 1.065–3.152, *p* = 0.0323), % inf > 15/25 (OR 3.904, CI 1.598–9.573, *p* = 0.0024), age > 60 years (OR 4.075, CI 1.791–8.559, *p* = 0.0004), Kirby index < 300 (OR 23.16, CI 3.625–247.3, *p* =  < 0.0001), Kirby index < 200 (OR 11.76, CI 4.691–28.26, *p* =  < 0.0001), sO_2_ < 90% (OR 2.619, CI 1.347–5.031, *p* = 0.0042), sO_2_ < 80% (OR 9.048, CI 3.746–21.41, *p* =  < 0.0001), CRP > 120 mg/dL (OR 2.611, CI 1.467–4.591, *p* = 0.0008), ferritin > 150 mg/dL (OR 91.38, CI 22.49–387.3, *p* =  < 0.0001), LDH > 211 U/L (OR 5.876, CI 1.811–19.26, *p* = 0.0021), LDH > 400 U/L (OR 2.730, CI 1.575–4.673, *p* = 0.0003), and AST > 70 U/L (OR 2.349, CI 1.060–5.056, *p* = 0.0330),

And finally, we found 15 markers associated with enhanced probability for mortality, which were creatinine > 1 mg/dL (OR 2.4868, CI 1.3698–4.5148, *p* = 0.0023), d-Dimer > 500 ng/mL (OR 3.272, CI 1.271–7.970, *p* = 0.0130), neutrophils > 7700 cells/µL (OR 2.645, CI 1.447–4.997, *p* = 0.0018), % inf > 15/25 (OR 80.35, CI 31.95–194.4, *p* =  < 0.0001), age > 60 years (OR 8, CI 2.22–28.9, *p* = 0.0191), MAP < 65 (OR 4.085, CI 1.016–18.04, *p* = 0.0451), Kirby index < 300 (OR 7.282, CI 1.141–78.42, *p* = 0.0284), Kirby index < 140 (OR 6.517, CI 2.361–16.10, *p* =  < 0.0001), sO_2_ < 90% (OR 0.3199, CI 16.24–0.6408, *p* = 0.0011), sO_2_ < 80% (OR 2.11, CI 1.168–3.951, *p* = 0.0143), CRP > 120 mg/dL (OR 4.546, CI 2.596–8.178, *p* =  < 0.0001), ferritin > 150 ng/mL (OR 8.571, CI 0.4919–147.4732, *p* = 0.0463), > 1 comorbidity (OR 2, CI 1.0824–3.9972, *p* = 0.0266), LDH > 211 U/L (OR 4.0854, CI 0.9290–17.9664, *p* = 0.0451) and LDH > 400 U/L (OR 3.338, CI 1.808–5.978, *p* =  < 0.0001).

**Figure 1:**
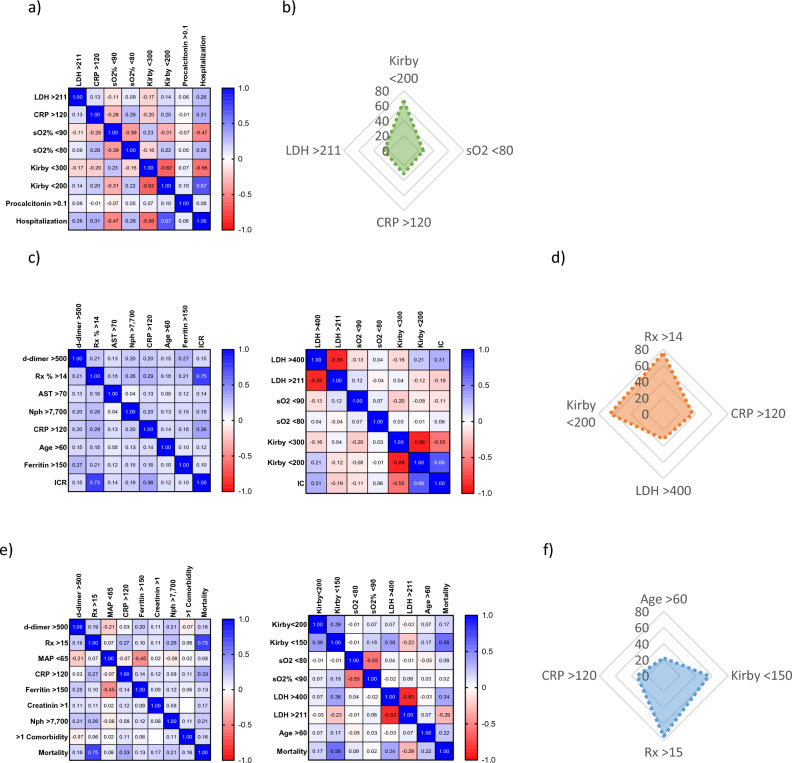
Mathematical functions for the association between biomarkers and outcomes. The biomarkers with significant odds ratios were used to calculate the Pearson’s correlations with HR (**a**), ICR (**c**) and mortality (**e**). Pearson’s indexes > 0.20 were considered to be highly associated, and their specific values were taken as functions to calculate the enhanced probability of HR (**b**), ICR (**d**) and mortality (**f**).

The markers with significant associations were plotted into a heat map and their Pearson’s correlation coefficient was calculated (Fig. 1a,c,e) and used to determine the relative weight, or mathematical function, of each variable into each of the three algorithms. Only four variables had a Pearson’s correlation index ≥ 0.20 in relation to each outcome, and thus were considered for the development of the algorithms, being Kirby < 200, LDH > 211, CRP > 120 and sO_2_ < 80 important for the prognostic of HR; Rx > 14, Kirby < 200, CRP > 120, and LDH > 400 for the prediction of ICR; as well as age > 60, Kirby < 150, CRP > 120 and Rx > 15 for mortality (Fig. 1b,d,f).

**Figure 2:**
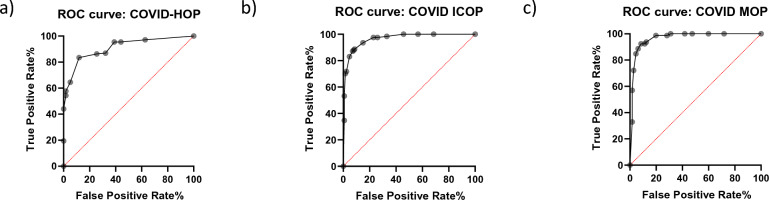
Sensitivity of the COVID-19 outcome prognostic scales. The area under the receiving operator characteristics curve was measured for the hospitalization (**a**), intensive care (**b**) and mortality outcome prognostic algorithms (**c**). *ROC* receiving operator characteristics, *AUROC* area under the ROC curve, *HOP* hospitalization-outcome prognostic, *ICOP* intensive care-outcome prognostic, *MOP* mortality-outcome prognostic.

To calculate the OP score for each patient, the Pearson’s index belonging to each variable (Fig. 1b,d,f) was added each time a particular patient presented an abnormal level of a particular marker, and then both control (outcome negative) and experimental (outcome positive) patients’ values were used to calculate the area under the ROC curve (AUROC). The COVID-hospitalization outcome prognostic (COVID-HOP) scale had an AUROC of 91% (CI 0.8725–0.9482 at 95%, *p* < 0.0001), and both the COVID-intensive care outcome prognostic (COVID-ICOP) and the COVID-mortality outcome prognostic (COVID-MOP) scales had an AUROC of 96% (CI 0.9448–0.9855 at 95%, *p* < 0.0001 and CI 0.9464–0.9872 at 95%, *p* < 0.0001, respectively) (Fig. 2).

The cutoff value for the COVID-HOP scale was found to be 52.7, while the COVID-ICOP was 113.1 and the COVID-MOP was 109 (Supp. Fig. S2). Thus, the complete algorithms with cutoff values were designed as detailed in Table 1, where the mathematical function of each marker, given by the Pearson’s correlation index, would add each time the patient presents levels that exceed the reference values of said marker, and if the scale’s cut-off value is exceeded by such sum, the patient would be considered at risk of either dying, needing regular hospitalization or intensive care.

**Table 1:** Algorithms for COVID-HOP, COVID-ICOP and COVID-MOP calculations.

	OP algorithms		
	COVID-HOP	COVID-ICOP	COVID-MOP
Kirby index	If < 200 add 67	If < 200 add 66	If < 150 add 67
Lactate dehydrogenase (LDH)	If > 211 add 26	If > 400 add 31	n.r
Oxigen saturation (sO_2_)	If < 80 add 26	n.r	n.r
C-reactive protein (CRP)	If > 120 add 31	If > 120 add 36	If > 120 add 30
Lung infiltration percentage (25 points scale on TAC)	n.r	If > 14 add 75	If > 15 add 75
Age	n.r	n.r	If > 60 add 22
	If sum ≥ 52.7, patient at risk	If sum ≥ 113.1, patient at risk	If sum ≥ 109, patient at risk

*OP* outcome prognostic, *HOP* hospitalization outcome prognostic, *ICOP* intensive care outcome prognostic, *MOP* mortality outcome prognostic, *n.r.* not-relevant.

Furthermore, the SE, SP, PPV, NPV (Table 2) and OR (Supp. Fig. S3) for each OP scale with the use of the respective cutoff values were calculated, finding that the COVID-HOP had a SE of 86%, SP of 74%, PPV of 90%, NPV of 94% and OR of 18.4 with a CI at 95% of 8.6–36, *p* ≤ 0.0001. On the other hand the COVID-ICOP had a SE of 87%, SP of 92%, PPV of 92%, NPV of 88% and OR of 88.5 with a CI at 95% of 35–191, *p* ≤ 0.0001. Finally, the COVID-MOP had a SE of 92%, SP of 91%, PPV of 82%, NPV of 96% and OR of 131 with a CI at 95% of 47–341, *p* ≤ 0.0001.

**Table 2:** Sensitivity, specificity, positive and negative predictive values for the outcome-prognostic scales.

Variable	Effect size	CI at 95%	p value
COVID-HOP			
Sensitivity	0.8629	0.8040–0.9061	0.0001
Specificity	0.7458	0.6220–0.8394	0.0001
Positive predictive value	0.9096	0.8563–0.9445	0.0001
Negative predictive value	0.6471	0.5284–0.7500	0.0001
COVID-ICUOP			
Sensitivity	0.8710	0.8006–0.9190	0.0001
Specificity	0.9291	0.8708–0.9623	0.0001
Positive predictive value	0.9231	0.8603–0.9590	0.0001
Negative predictive value	0.8806	0.8148–0.9252	0.0001
COVID-MOP			
Sensitivity	0.9241	0.8440–0.9647	0.0001
Specificity	0.9153	0.8649–0.9480	0.0001
Positive predictive value	0.8295	0.7376–0.8939	0.0001
Negative predictive value	0.9643	0.9243–0.9835	0.0001

*HOP* hospitalization-outcome prognostic, *ICOP* intensive care outcome-prognostic, *MOP* mortality outcome-prognostic.

Furthermore, 167 patients’ records belonging to a different health center and that were not used to calculate the algorithms, were retrospectively studied to perform a validation of the SE, SP, PPV and NPV. Only 26% of these patients (32 individuals) were vaccinated against the coronavirus. The results for the MOP algorithm showed no variation in the second population tested, while the ICOP scale exhibited only minimal variation. In regards to the HOP algorithm, the specificity was considerably reduced (0.74 in the creation of the algorithm, 0.36 in the test of accuracy), but the other parameters remained without significant changes (Table 3). Finally, an online version of the algorithms was developed to facilitate its use, and can be found at: http://benepachuca.no-ip.org/covid/index.php

**Table 3:** Validation experiment for the HOP, ICOP and MOP algorithms.

Variable	Effect size	CI at 95%	p value
COVID-HOP			
Sensitivity	0.9398	0.8666–0.9740	0.0001
Specificity	0.3684	0.2338–0.5272	0.0001
Positive predictive value	0.7647	0.6737–0.8365	0.0001
Negative predictive value	0.7368	0.5121–0.8819	0.0001
COVID-ICOP			
Sensitivity	0.9365	0.8478–0.9750	0.0001
Specificity	0.8596	0.7468–0.9271	0.0001
Positive predictive value	0.8806	0.7817–0.9382	0.0001
Negative predictive value	0.9245	0.8214–0.9703	0.0001
COVID-MOP			
Sensitivity	0.9241	0.8440–0.9647	0.0001
Specificity	0.9153	0.8649–0.9480	0.0001
Positive predictive value	0.8295	0.7376–0.8939	0.0001
Negative predictive value	0.9643	0.9243–0.9835	0.0001

## Discussion

In the present research we assessed the degree of correlation of 16 biomarkers (three of them with 2 different reference limits) with three different outcomes (the future need for hospitalization and/or intensive care as well as the enhanced probability of mortality) by calculating the odds ratio, revealing six markers associated with the first outcome, 13 with the second, and 15 with the last. Nonetheless, when the data was binary transformed and analyzed by the means of a Pearson’s correlation, only four markers were found to be associated with each marker: (i) Kirby < 200, LDH > 211, sO2 < 80 and CRP > 120 were highly associated with the requirement for hospitalization; Rx > 14, Kirby < 200, CRP > 120, and LDH > 400 were strongly related to the requirement for intensive care; and finally, age > 60, CRP > 120, Rx > 15 and Kirby < 150 correlated with a high mortality.

We then developed three different algorithms, all of them based on adding the Pearson’s correlation index for the markers that were relevant to each outcome, every time a patient developed pathological levels of a particular molecule. Interestingly, when calculating the mathematical functions in biomedical sciences a common approach is to perform a nomogram^27^, because of the underlying convenience of such technique. Nonetheless, the precision of such a graphic tool is not remarkable. In these circumstances, the addition of the Pearson’s coefficients helped to develop a series of tools with enhanced sensitivity, as the COVID-HOP, COVID-ICOP, and COVID-MOP algorithms showed a sensitivity over 90% in each case.

Currently many meta-analysis^28–33^ studying the risk factors and biomarkers for prediction of COVID-19 outcomes are available, but these are primarily based on cohort studies that are only representative of the Asian population, with minimal involvement of other genetic backgrounds. In this instance, the aforementioned studies’ results reflect an enhanced degree of similarity for all the clinical and laboratory findings. Nonetheless, when different populations are studied the level of association of some biomarkers with the disease outcomes varies^34,35^, in such a way that the evaluation of prognostic markers in different populations may be of paramount importance to enhance the sensitivity and specificity of a prognostic tool. In accordance to this line of thought, here we present results derived from the analysis of a Mexican population, that reflect key differences in the association of prognostic markers with outcomes of enhanced pathology, in which the absence of a positive correlation between comorbidities and the worsening of COVID-19 stands out.

However, in a validation experiment we observed that the degree of SE, SP, PPV, and NPV varied only in a slight manner, despite of using data belonging to patients from a different hospital, and with a quarter part of them having been vaccinated (a condition that was not present in the patients that provided the data for the elaboration of the algorithm). In any way, further research is needed to confirm if such homogeneity is paralleled in an international cohort. If the present tools does not possess enhanced precision, the development of specific algorithms for each region may be a viable option.

Finally, the chest CT evaluation is made subjectively according to the physician’s appreciation, which could impair the results of the prognostics for enhanced mortality and intensive care requirement, as this marker has an increased weight into these algorithms. Nonetheless, excellent new technologies appear to be emerging on the field, in which such evaluation is made accurately^36,37^, and its widespread use may be helpful in the homologation of prognostic criteria.

Overall, these results show the development of three tools that may aid in the administration of hospital resources, including regular hospital beds, intensive care unit beds, and drugs. Such technology may be of enhanced utility in the context of the pandemic waves, which are expected to be a common occurrence in the coming years^38^, especially since no vaccine formula has been proven to produce sterilizing immunoglobulin titers^39^. In fact, expert committees have agreed that healthcare digital innovations are both lacking^40^ and necessary^41^ to enhance hospital resiliency, thus making necessary the development of this kind of tools.

## Supplementary Information

Supplementary Information.

## Data Availability

Data is available upon reasonable request to the corresponding author Alberto Navarrete Peón at investigacion@benepachuca.com.

## Abbreviations

ICU: Intensive care unit
ICR: Intensive care requirement
HR: Hospitalization requirement
OP: Outcome prognostic
ROC: Receiver operator characteristics
SE: Sensitivity
SP: Specificity
PPV: Positive predictive value
NPV: Negative predictive value
IC: Intensive care
%inf: Extension of the inflammatory infiltrate of the lungs measured by tomography
CRP: C-reactive protein
LDH: Lactate dehydrogenase
MAP: Medium arterial pressure
AST: Aspartate aminotransferase
sO2: Oxygen saturation
AUROC: Area under the ROC curve
HOP: Hospitalization-outcome prognostic
ICOP: Intensive care-outcome prognostic
MOP: Mortality-outcome prognostic
