# Supplementary material for: A novel scale based on biomarkers associated with COVID-19 severity can predict the need for hospitalization and intensive care, as well as enhanced probabilities for mortality
Source: Sci Rep. 2023 Jun 4;13:9064. doi: 10.1038/s41598-023-30913-4 (PMC10239539; doi:10.1038/s41598-023-30913-4)
Supplement: Supplementary file 1 — Supplementary Information. [file 41598_2023_30913_MOESM1_ESM.docx]

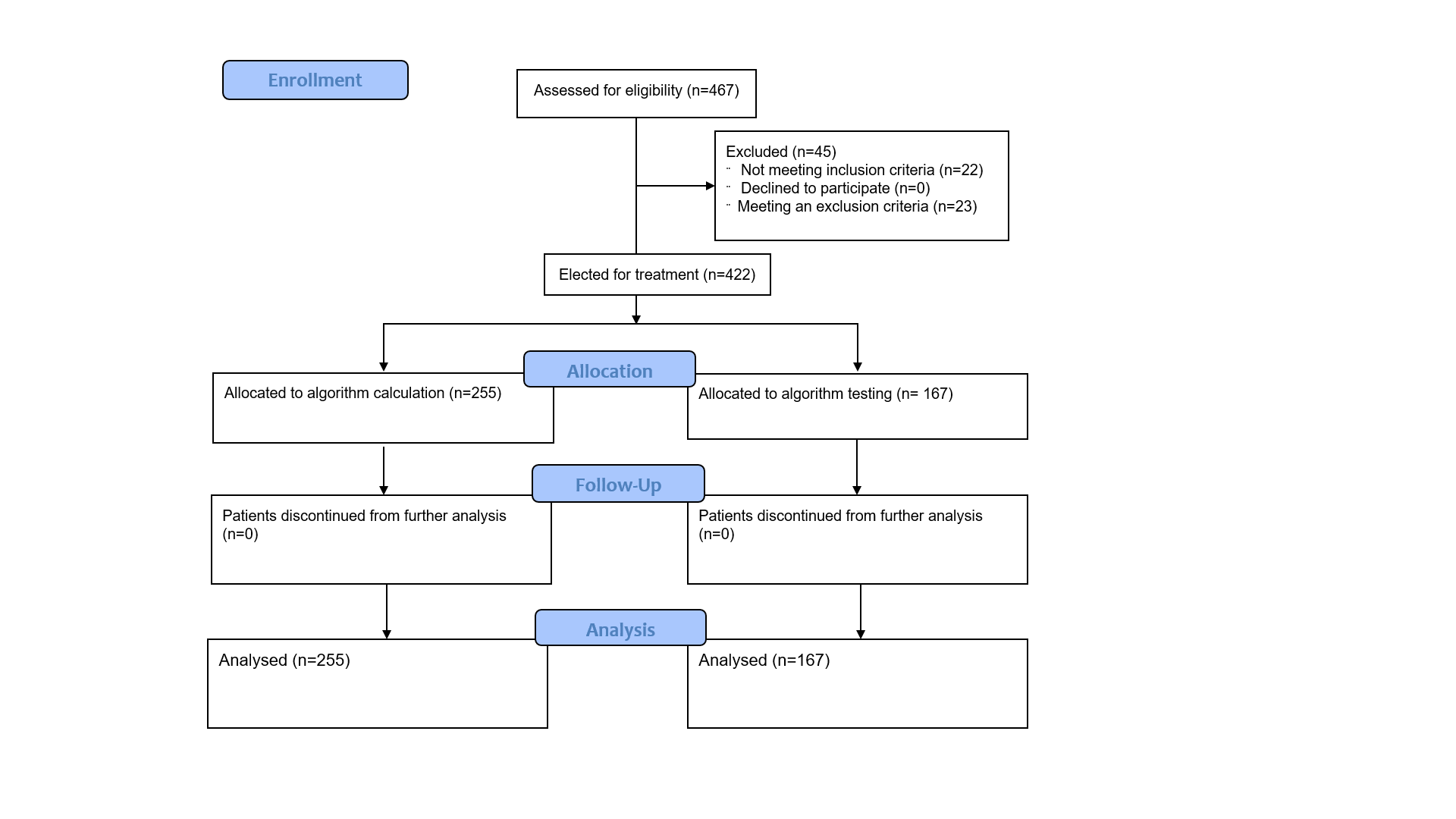


Supplementary figure 1. Screening and eligibility of clinical records. A total of 467 clinical files of SARS-CoV-2-positive patients were identified in two health centers in México. 422 met the inclusion criteria, while 45 met one or more exclusion criteria. Finally, 422 clinical files were included in the study.

Supplementary table 1. Association of different biomarkers with hospitalization requirement.

| Biomarker | Odds ratio | 95% confidence interval | *p* value |
| --- | --- | --- | --- |
| Kirby index <300 | 5.58 | 1.95-15.97 | 0.0010 |
| Kirby index <200 | 34.16 | 15.10 to 70.99 | 0.0001 |
| sO_2_ <90% | 2.15 | 1.138-3.930 | 0.0133 |
| sO_2_ <80% | 3.04 | 1.415-6.833 | 0.0043 |
| CRP >120 mg/dL | 2.95 | 1.439-5.838 | 0.0021 |
| LDH >400 U/L | 2.6 | 1.393-4.982 | 0.0034 |
| LDH >211 U/L | 2.813 | 0.172-6.820 | 0.237 |
| Creatinin >1 mg/dL | 1.965 | 0.9523-4.137 | 0.0652 |
| d-Dimer >500 ng/mL | 1.403 | 0.6358-3.101 | 0.3911 |
| MAP <65 | 0.1791-infinity |  |  |
| Lymphocytes <1000 cells/µL | 1.259 | 0.6909-2.245 | 0.4482 |
| Glucose >200 mg/dL | 1.101 | 0.5239-2.237 | 0.7956 |
| Age >60 years | 1.346 | 0.6792-2.668 | 0.4164 |
| >2 comorbidities | 1.311 | 0.6722-2.613 | 0.4354 |
| One comorbidity | 1.140 | 0.6396-2.075 | 0.6673 |
| Neutrophils >7700 cells/µL | 1.672 | 0.9005-3.097 | 0.0917 |
| % inf >15/25 | 1.160 | 0.3329-3.900 | 0.8218 |
| Ferritin >150 ng/mL | 1.772 | 0.4695-6.645 | 0.4255 |
| AST >70 U/L | 0.9281 | 0.3942 to 2.394 | 0.8729 |

Abbreviations: sO_2_, oxygen saturation; CRP, C-reactive protein; LDH, lactate dehydrogenase; MAP, medium arterial pressure; %inf, percentage of inflammatory infiltration in the lungs; AST, aspartate aminotransferase.

Supplementary table 2. Association of different biomarkers with intensive care requirement.

| Biomarker | Odds ratio | 95% confidence interval | *p* value |
| --- | --- | --- | --- |
| d-Dimer >500 ng/mL | 2.411 | 1.160 to 5.017 | 0.0179 |
| Neutrophils >7700 cells/µL | 1.807 | 1.065 to 3.152 | 0.0323 |
| % inf >15/25 | 3.904 | 1.598 to 9.573 | 0.0024 |
| Age >60 years | 4.075 | 1.791 to 8.559 | 0.0004 |
| Kirby <300 | 23.16 | 3.625 to 247.3 | <0.0001 |
| Kirby <200 | 11.76 | 4.691 to 28.26 | <0.0001 |
| sO_2_ <90% | 2.619 | 1.347 to 5.031 | 0.0042 |
| sO_2_ <80% | 9.048 | 3.746 to 21.41 | <0.0001 |
| CRP >120mg/dL | 2.611 | 1.467 to 4.591 | 0.0008 |
| Ferritin >150 ng/mL | 91.38 | 22.49 to 387.3 | <0.0001 |
| LDH >211 U/L | 5.876 | 1.811 to 19.26 | 0.0021 |
| LDH >400 U/L | 2.730 | 1.575 to 4.673 | 0.0003 |
| AST >70 U/L | 2.349 | 1.060 to 5.056 | 0.0330 |
| Creatinin >1 mg/dL | 1.160 | 0.6531 to 2.039 | 0.6002 |
| MAP <65 | 2.756 | 0.3102 to 40.49 | 0.3954 |
| Procalcitonin >0.1 | 2.003 | 0.7814 to 5.058 | 0.1315 |
| Glucose >200 mg/dL | 0.9332 | 0.5191 to 1.739 | 0.8221 |
| Lymphocytes <1000 cells/µL | 1.306 | 0.7920 to 2.168 | 0.3022 |
| One comorbidity | 1.044 | 0.6378 to 1.713 | 0.8657 |
| >1 comorbidities | 1.171 | 0.6708 to 2.031 | 0.5851 |

Abbreviations: sO_2_, oxygen saturation; CRP, C-reactive protein; LDH, lactate dehydrogenase; MAP, medium arterial pressure; %inf, percentage of inflammatory infiltration in the lungs; AST, aspartate aminotransferase.

Supplementary table 3. Association of different biomarkers with mortality.

| Biomarker | Odds ratio | 95% confidence interval | *p* value |
| --- | --- | --- | --- |
| Creatinin >1 mg/dL | 2.4868 | 1.3698 to 4.5148 | 0.0023 |
| d-Dimer >500 | 3.272 | 1.271 to 7.970 | 0.0130 |
| Neutrophils >7700 cells/µL | 2.645 | 1.447 to 4.997 | 0.0018 |
| % inf >15/25 | 80.35 | 31.95 to 194.4 | <0.0001 |
| Age >60 years | 8 | 2.22 to 28.9 | 0.0191 |
| MAP <65 | 4.085 | 1.016 to 18.04 | 0.0451 |
| Kirby <300 | 7.282 | 1.141 to 78.42 | 0.0284 |
| Kirby <140 | 6.517 | 2.361 to 16.10 | <0.0001 |
| sO_2_ <90% | 0.3199 | 0.1624 to 0.6408 | 0.0011 |
| sO_2_ <80% | 2.11 | 1.168 to 3.951 | 0.0143 |
| CRP >120mg/dL | 4.546 | 2.596 to 8.178 | <0.0001 |
| Ferritin >150 ng/mL | 8.517 | 0.4919 to 147.4732 | 0.0463 |
| > 1 comorbidities | 2 | 1.0824 to 3.9972 | 0.0266 |
| LDH >211 U/L | 4.0854 | 0.9290 to 17.9664 | 0.0451 |
| LDH >400 U/L | 3.338 | 1.808 to 5.978 | <0.0001 |
| Glucose >200 mg/dL | 1.3771 | 0.7421 to 2.5554 | 0.3092 |
| Lymphocytes <1000 cells/µL | 0.9176 | 0.5347 to 1.5746 | 0.7548 |
| AST >70 U/L | 1.5 | 0.6605 to 3.4068 | 0.3303 |
| One Comorbidity | 1.5 | 0.8142 to 2.8699 | 0.1853 |
| Procalcitonin >0.05 ng/ml | 1.2 | 0.6649 to 1.6722 | 0.7975 |

Abbreviations: sO_2_, oxygen saturation; CRP, C-reactive protein; LDH, lactate dehydrogenase; MAP, medium arterial pressure; %inf, percentage of inflammatory infiltration in the lungs; AST, aspartate aminotransferase.


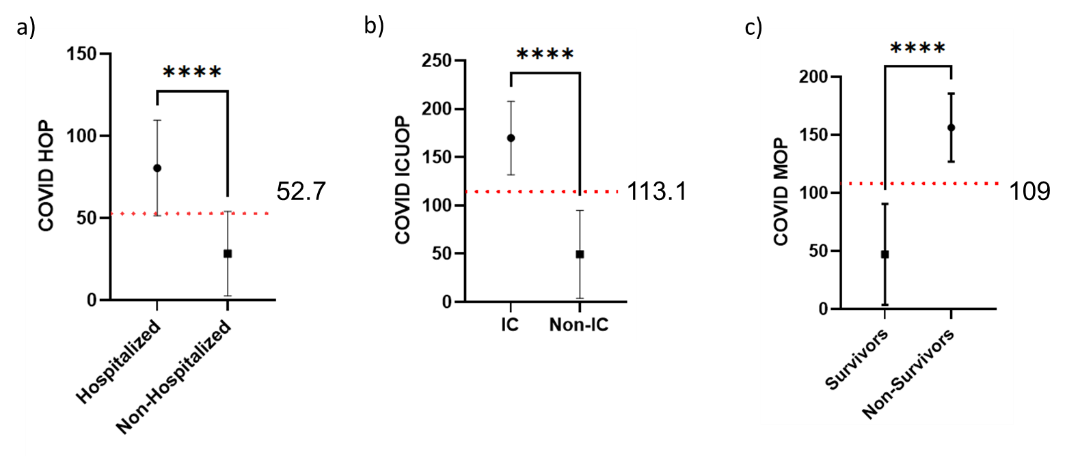


Supplementary figure 2. Cutoff values for the COVID HOP, ICOP and MOP scales. The cutoff value for the COVID HOP (a), COVID ICOP (b) and COVID MOP (c) prognostic scales was found by adding the standard deviation to the mean of the control group, subtracting the standard deviation to the mean of the affected group and finding the middle point between the two cyphers. Abbreviations: HOP, hospitalization outcome prognostic; ICOP, intensive care outcome prognostic; MOP, mortality outcome prognostic.


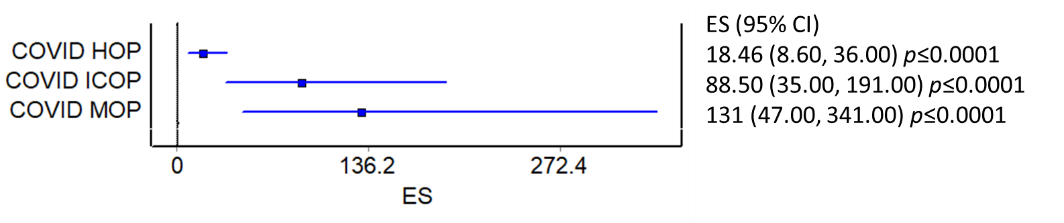


Supplementary figure 3. Odds ratios for the COVID-OP scales. The effect size (ES) for the three scales was calculated as an odds ratio, which was complimented by the 95% confidence interval. Abbreviations: HOP, hospitalization outcome prognostic; ICOP, intensive care outcome prognostic; MOP, mortality outcome prognostic.
